# Giant Intradiploic Epidermoid Cyst of the Occipital Bone with Posterior Fossa Compression: A Case Report

**DOI:** 10.1055/a-2804-2307

**Published:** 2026-03-17

**Authors:** Halit Alioglu, Uğur Can Yılmaz, Mahmoud Osama, Zuhal Kuş Silav, Bulent Guclu

**Affiliations:** 1 Department of Neurosurgery, University of Health Sciences, Dr. Lutfi Kirdar Kartal Training and Research Hospital, Istanbul, Türkiye; 2 Department of Neurosurgery, Acıbadem University Hospital, Istanbul, Türkiye; 3 Department of Pathology, University of Health Sciences, Dr. Lutfi Kirdar Kartal Training and Research Hospital, Istanbul, Türkiye

**Keywords:** epidermoid cyst, intradiploic, occipital bone, posterior fossa, gross-total excision

## Abstract

**Background:**

Intradiploic epidermoid cysts are rare lesions that account for a small fraction of intracranial tumors with occasional malignant transformation. Occipital involvement is particularly uncommon, and lesions may reach giant dimensions with a risk fossa compression.

**Case Presentation:**

A 60-year-old woman presented with a 12-month history of progressive headache and vertigo. Magnetic resonance imaging (MRI) revealed a large, diffusion-restricting extra-axial mass arising from the occipital bone and extending into the posterior fossa. She underwent a posterior fossa craniotomy with gross-total resection while preserving dural integrity. Histopathology confirmed an epidermoid cyst. The postoperative course was uneventful, and follow-up MRI showed complete resection with no recurrence.

**Conclusion:**

Giant intradiploic epidermoid cysts of the occipital bone, though histologically benign, can cause extensive bone destruction and significant posterior fossa mass effect. Accurate preoperative imaging and meticulous surgical planning are critical to achieving safe gross-total excision and favorable outcomes, particularly in atypical or extensive presentations.

## Introduction

Intracranial epidermoid cysts, first described by Müller in 1838, are rare, slow-growing tumors and represent 0.2% to 1.8% of all intracranial tumors. 1 2 They usually arise in the cerebellopontine angle or parasellar region, whereas localization within the calvarial diploë is distinctly uncommon. 3 4 Primary intradiploic epidermoid cysts are thought to result from ectodermal cell entrapment during neural tube closure. 5 Fewer than 250 cases have been reported, with the occipital bone among the least frequent sites. 6 When large, these lesions may extend intra- and extracranially, producing posterior fossa compression with headache, cerebellar ataxia, dizziness, or cranial nerve dysfunction. 7 Although histologically benign, they may reach “giant” dimensions (>5 cm) and can rarely undergo malignant transformation. 8 Diffusion-weighted imaging (DWI) is valuable in distinguishing epidermoid cysts from other cystic lesions, and gross-total excision with capsule removal remains the treatment of choice. 9 10 The first giant posterior fossa intradiploic epidermoid cyst was reported by Rengachary et al. in 1978, with subsequent reports refining understanding of their clinical course and surgical management. 11 12 13 14 To our knowledge, bilateral occipital involvement of this size is exceptionally rare. Herein, we describe such a case causing posterior fossa compression and discuss the lessons it provides.

## Case Presentation

A 60-year-old woman presented with a 12-month history of progressive headache and vertigo, with recent exacerbation over 1 month. Neurological examination was normal on admission.

**Fig. 1:**
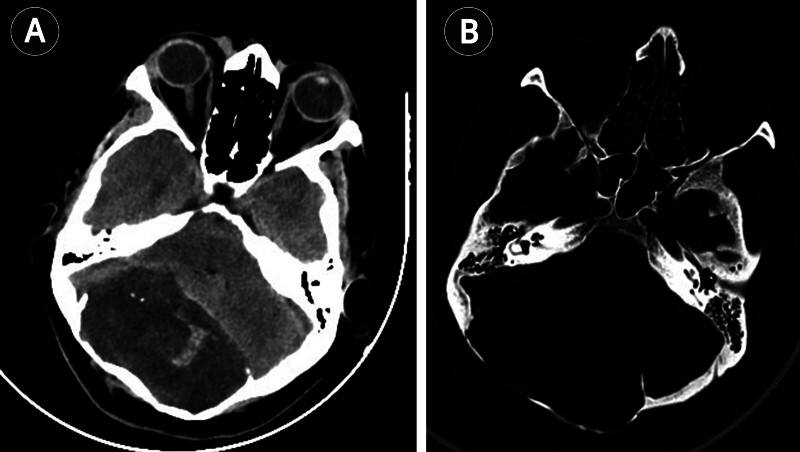
Axial non-contrast CT. ( **A** ) Well-demarcated hypodense lobulated mass filling the posterior fossa, causing marked compression of the fourth ventricle and brainstem. ( **B** ) Bone window showing intradiploic extension with pronounced thinning and erosion of both inner and outer tables of the occipital bone. CT, computed tomography.

**Fig. 2:**
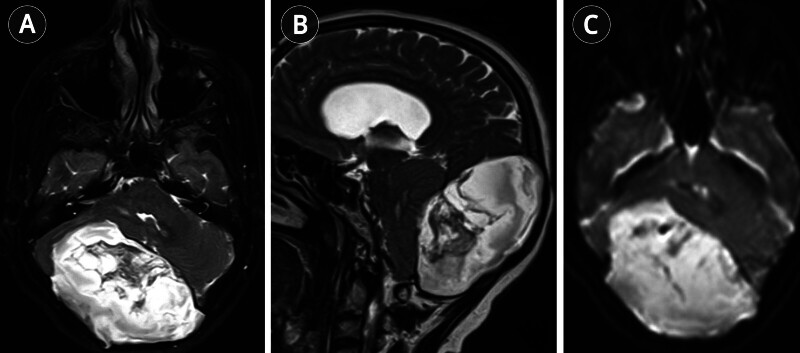
Preoperative MRI of the brain. ( **A** ) Axial T2-weighted image demonstrating a heterogeneously hyperintense mass in the posterior fossa compressing the fourth ventricle. ( **B** ) Sagittal T2-weighted image showing prominent mass effect on the brainstem with effacement of the fourth ventricle. ( **C** ) Diffusion-weighted image revealing uniform diffusion restriction throughout the lesion, consistent with an epidermoid cyst. MRI, magnetic resonance imaging.

**Fig. 3:**
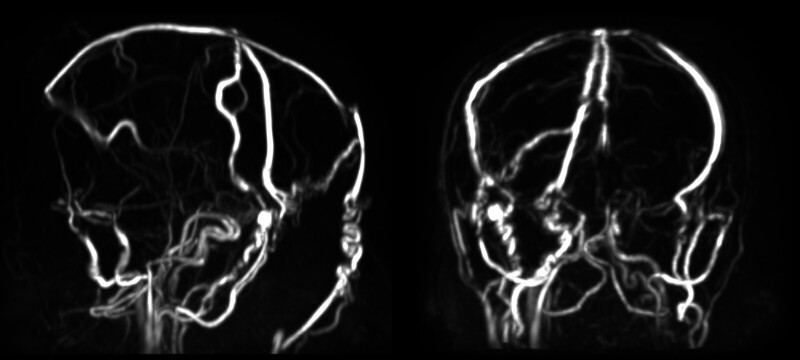
MR venography demonstrates marked compression and functional occlusion of the superior sagittal sinus and bilateral transverse sinuses, with extensive collateral venous drainage pathways, consistent with chronic extrinsic venous sinus compression rather than acute thrombosis. MR, magnetic resonance.

**Fig. 4:**
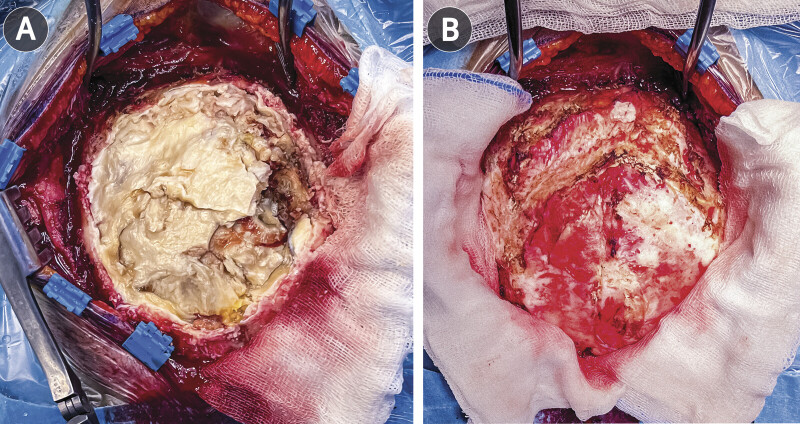
Intraoperative photographs. ( **A** ) Exposure of the lesion showing pearly keratinous material filling the intradiploic cavity. ( **B** ) Surgical field after complete resection, demonstrating intact underlying dura.

An emergent computed tomography (CT) was obtained, which revealed bilateral occipital diploic expansion with thinning of the outer table and scalloping and erosion of the inner table. There was significant posterior fossa mass effect with compression of the brainstem and fourth ventricle ( Fig. 1 ). Magnetic resonance imaging (MRI) of the brain demonstrated a large 9.5 × 9.5 × 6 cm extra-axial posterior fossa mass with heterogeneous FLAIR signal, serpiginous T1 hyperintensity, and mixed T2 signal ( Fig. 2 ). The lesion extended through the diploë, involving both the right and left occipital bones. Restricted diffusion was uniform. Magnetic resonance venography (MRV) revealed severe compression with functional occlusion of the superior sagittal and bilateral transverse sinuses ( Fig. 3 ). The patient underwent a posterior fossa craniotomy in the prone Concorde position with the head secured in a Mayfield clamp. A midline occipital incision was made over the region of maximal diploic expansion, and subperiosteal dissection revealed thinned, moth-eaten bone. On elevating the tailored occipital craniotomy flap, areas of full-thickness diploic erosion were encountered with extrusion of pearly, waxy material ( Fig. 4A ). The cyst contents were evacuated using suction and ring curettes, with repeated saline irrigation to reduce the risk of postoperative chemical meningitis. Following decompression, the thick whitish capsule was identified and dissected circumferentially under the operating microscope. It was broadly adherent to the inner table but separated cleanly from the dura, which remained intact with no CSF egress. Gross-total resection was achieved while preserving dural integrity ( Fig. 4B ). Hemostasis was secured with bone wax over diploic channels, and the bone flap was replaced and fixed with low-profile plates. There were no intraoperative complications or venous sinus injuries.

**Fig. 5:**
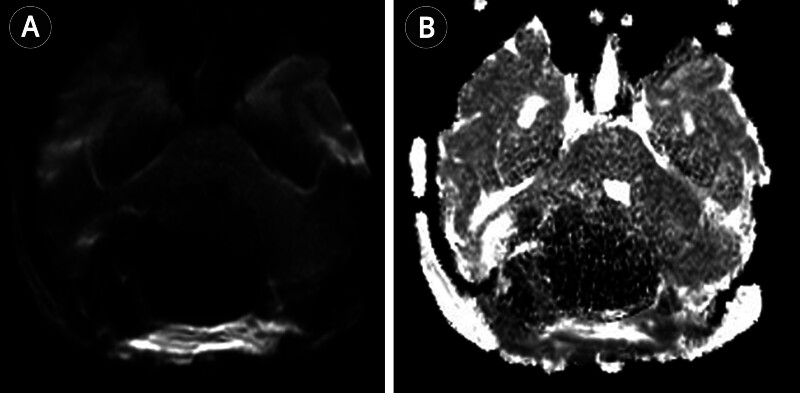
Postoperative DWI ( **A** ) and ADC map ( **B** ) show no residual diffusion restriction within the resection bed, consistent with complete resection and substantial reduction in mass effect. ADC, apparent diffusion coefficient; DWI, diffusion-weighted imaging.

**Fig. 6:**
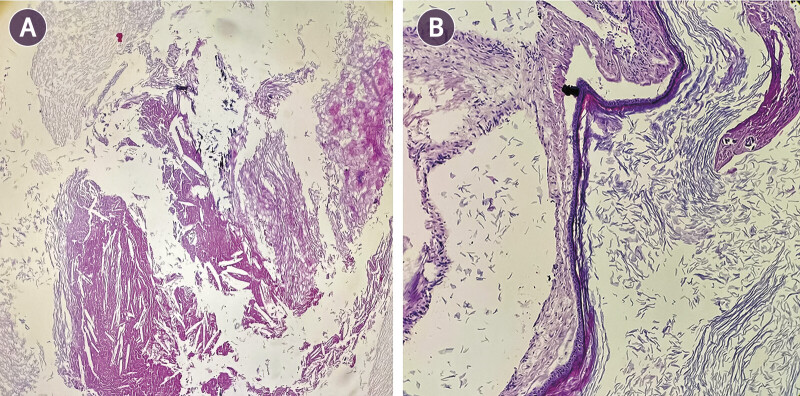
Histopathological examination of the biopsy. ( **A** ) Cystic lesion composed of laminated keratinous debris, accompanied by cholesterol clefts and focal dystrophic calcifications (hematoxylin and eosin, 40× HPF). ( **B** ) Thin cyst wall lined by pseudostratified squamous epithelium with laminated keratin debris in the cyst lumen (hematoxylin and eosin, 100× HPF). HPF, high-power field.

Postoperative MRI confirmed complete resection of the lesion with significant reduction in mass effect on the brainstem and basal cisterns ( Fig. 5 ). Histopathological examination showed a cyst lined by keratinizing stratified squamous epithelium, consistent with an epidermoid cyst ( Fig. 6 ). The patient recovered uneventfully and was discharged on postoperative day seven without deficits. At the 5-month follow-up, MRI demonstrated no recurrence, and the patient remained symptom-free.

## Discussion

The present case highlights the nature and behavior of the intradiploic epidermoid cyst, which is a slow-growing, histologically benign tumor, with indolent expansion producing profound clinical consequences when situated in the posterior fossa as the lesion extended across both occipital bones and caused posterior fossa compression. The occipital bone is one of the least common sites for intradiploic epidermoid cysts. 15 Rengachary et al reported the first giant posterior fossa lesion in 1978, measuring 13 cm, in the pre-MRI era. 11 Since then, several reports have confirmed that these lesions may achieve considerable dimensions before diagnosis, owing to their slow growth and non-specific symptoms. 4 16 The most frequent complaints include headache, cerebellar ataxia, localized swelling, dizziness, or visual disturbances, while cranial nerve deficits are less frequent and usually appear in advanced cases. 6 7 15 Our patient presented with chronic headaches and vertigo without neurological deficits, consistent with the often subtle clinical profile of these tumors. Radiological evaluation remains pivotal; MRI demonstrates heterogeneous signal characteristics with uniform restricted diffusion, considered pathognomonic for epidermoid cysts. 9 Our case exhibited these features, corroborating previous reports. 13 14 CT is complementary, showing expansion of the diploë with thinning or erosion of the inner and outer tables. However, CT may be the first investigation required in cases of emergency presentation, as was the case in our instance. In our patient, extensive bony destruction was seen bilaterally, which has been described only in a limited number of prior cases. 12 Given the extensive involvement of the superior sagittal and bilateral transverse sinuses, careful preoperative assessment of venous outflow was essential. MRV demonstrated severe sinus compression with markedly reduced flow in the superior sagittal and bilateral transverse sinuses, accompanied by prominent collateral venous channels, suggesting chronic extrinsic compression rather than acute thrombosis ( Fig. 3 ). This finding supported a surgical strategy focused on decompression without direct manipulation of the venous sinuses. Intraoperatively, meticulous subperiosteal dissection and microsurgical techniques were employed to avoid dural violation and venous injury. Preservation of dural integrity was prioritized to minimize the risk of venous outflow compromise, and no signs of venous infarction or raised intracranial pressure were observed postoperatively. Although uniform diffusion restriction on MRI is a well-recognized and highly characteristic feature of epidermoid cysts, giant intradiploic lesions with extensive bone destruction may still raise a broad differential diagnosis. 9 Calvarial metastasis and plasmacytoma were excluded based on the well-demarcated, non-enhancing nature of the lesion, uniform diffusion restriction, and the characteristic pearly keratinous contents encountered intraoperatively. These features, together with histopathological confirmation, supported the final diagnosis of an intradiploic epidermoid cyst. Surgical management aims for gross-total excision, including removal of the cyst capsule, to minimize the risk of recurrence. 10 However, adherence to critical structures such as dura or venous sinuses often complicates complete removal. In our case, the capsule was carefully dissected from the dura, and gross total resection was achieved, similar to the successful outcomes reported by Choo et al and Oommen et al. 17 18 Importantly, bilateral bony involvement posed an additional surgical challenge, but resection was feasible without dural breach or venous injury. Although prognosis is generally favorable after complete excision, rare cases of malignant transformation into squamous cell carcinoma have been reported. 8 19 This emphasizes the necessity of continued follow-up even after gross-total resection. In our case, follow-up MRI at 5 months demonstrated no recurrence, but long-term surveillance remains essential. Taken together, our case underscores three key observations: First, bilateral intradiploic involvement of the occipital bone represents an exceptionally rare presentation, broadening the clinical spectrum of posterior fossa epidermoid cysts; second, modern neuroimaging allows accurate preoperative diagnosis, which is critical in surgical planning; and third, gross-total resection remains the gold standard, yet surgical success depends on meticulous technique when the lesion abuts critical venous sinuses. Future accumulation of such cases will help refine management strategies and clarify long-term outcomes in this uncommon entity.

## Conclusion

Intradiploic epidermoid cysts of the posterior fossa are rare entities that can remain clinically silent until reaching considerable size. Our case of a giant bilateral occipital intradiploic epidermoid cyst underscores the potential for extensive bony destruction and posterior fossa compression despite the benign histology. Preoperative imaging, particularly diffusion-weighted MRI, is critical for diagnosis and surgical planning. Gross-total resection remains the treatment of choice, offering favorable outcomes when achieved without injury to critical venous structures. This report adds to the limited literature on posterior fossa intradiploic epidermoid cysts and emphasizes the importance of recognizing atypical and bilateral presentations.
